# Editorial: Recent advancements in RNA-based and targeted therapeutics

**DOI:** 10.3389/fgene.2025.1603498

**Published:** 2025-04-08

**Authors:** Yiming Meng, Viola Bianca Serio, Yuquan Tong, Tao Huang, Elisa Frullanti, Maria Palmieri

**Affiliations:** ^1^ Department of Central Laboratory, Cancer Hospital of Dalian University of Technology, Liaoning Cancer Hospital & Institute, Shenyang, Liaoning, China; ^2^ Cancer Genomics and Systems Biology Lab, Department of Medical Biotechnologies, University of Siena, Siena, Italy; ^3^ Department of Medical Biotechnologies, Med Biotech Hub and Competence Centre, University of Siena, Siena, Italy; ^4^ Department of Chemistry, The Scripps Research Institute La Jolla, Jupiter, FL, United States; ^5^ Shanghai Institute of Nutrition and Health, Chinese Academy of Sciences (CAS), Shanghai, China

**Keywords:** RNA, therapeutics, drug discovery, drug delivery, oligonucleotides

## Introduction

In the early 1980s, the development and application of antisense oligonucleotides that could specifically bind to messenger RNA (mRNA) and inhibit protein synthesis represented a groundbreaking advancement in molecular biology. These synthetic, single-stranded nucleic acid sequences were designed to complement target mRNA strands, thereby blocking their translation into proteins. This innovative approach not only provided a powerful tool for studying gene expression and function but also promoted the rapid advances of RNA-based therapeutics (Zhu, 2022).

RNA epigenetic modifications have been uncovered as not only an intermediary structure between DNA and protein or an effector molecule, but it plays crucial roles in post-transcriptional gene regulation. As happens with targeted therapy in DNA blocking specific molecular signals that promote tumor growth and improve treatment efficacy reducing side effects (Rina et al., 2024), the functional and structural diversity of RNA promotes its applications in therapeutics. Several RNA-based medications have been approved for clinical use, while others are still under investigation or preclinical trials.

Numerous studies have established the critical role of RNA in a broad range of diseases including COVID-19, neurological diseases, and various cancers.

RNA-based gene therapy requires therapeutic RNA to function inside target cells without eliciting unwanted immune responses. RNA can be ferried into cells using non-viral drug delivery systems, which circumvent the limitations of viral delivery vectors (Paunovska et al., 2022).

Therefore, targeting RNA harbors profound potential to expand the “druggability” of the human genome. On the other hand, the development of small interfering RNA (siRNA), RNA interferences (RNAi), and mRNA vaccines have shown RNA as not only disease targets but also therapeutics themselves. Collectively, the complex yet important role of RNA demands further studies from multiple aspects.

This Research Topic focused on the recent advances in the field of RNA-related research with a highlight on translational values to accelerate the development of RNA-based and RNA-targeted biomedical applications in the field of epigenomics and epigenetics.

## Advances in RNA research

In this Research Topic, five original research papers have been published regarding new scientific advances in the RNA research field. In particular, Zhang et al. uncovered the miRNA expression profiles in chronic intermittent hypoxia (CIH)-induced WAT dysfunction mice. They established apolipoprotein deficient (ApoE−/−) mice CIH model and traced differential expressed miRNAs (DEmiRs) through miRNA-seq technology discovering 31 DEmiRs in the ApoE−/−mice model of CIH. Their findings may play a major role in explaining the pathophysiology mechanisms of WAT dysfunction induced by obstructive sleep apnea.

Liu et al. highlighted the trends and frontiers of RNA methylation in cancer over the past 10 years. They searched publications on RNA methylation in cancer and were retrieved from the Web of Science Core Collection database. VOSviewer, CiteSpace, and Bibliometrix were used to conduct bibliometric and visualization analysis of countries, institutions, authors, journals, and keywords relevant to this field. Their research provides a valuable reference for researchers in this field.

The aim of Li et al. study is to investigate the effect of m7G-related lncRNA on ovarian cancer in terms of instruction prognosis and immunotherapy. They attained a prognostic risk model which is excellent in predicting the prognostic survival of ovarian cancer patients as well as existing as an independent prognostic factor. Moreover, the model has certain relevance in the immune cells and functions between high and low risk groups, and simultaneously, the signature has the role of guiding the option of immunotherapy and chemotherapeutic drugs.

Wei et al. studied the disulfidptosis as a newly recognized form of regulated cell death that has been linked to cancer progression and prognosis. A total of eight core disulfidptosis-related lncRNAs were obtained to construct a prognostic model for ovarian cancer, categorizing all patients into low-risk and high-risk groups. They found a reliable and novel prognostic model with a disulfidptosis-related lncRNAs cluster for ovarian cancer, providing a foundation for further researches in the management of this disease.

Last but not the least, Li et al. studied a model based on plasma cell markers in hepatocellular carcinoma (HCC) through single-cell sequencing analysis. The identification of the prognostic risk model demonstrated exceptional predictive accuracy for overall survival and exhibited varying sensitivities to immunotherapy and chemotherapy among patients with HCC. Their data demonstrated that the risk score stood as an independent prognostic predictor and the nomogram results further affirmed its strong prognostic capability. This study reveals the heterogeneity of Tumor infiltrating B lymphocytes and provides a prognostic risk model based on plasma cell markers in HCC, which could prove valuable in predicting prognosis and guiding the choice of suitable therapies for patients with HCC.

## Conclusion

In conclusion, these studies demonstrate that RNA represents a novel and promising therapeutic target, with potential applications in a wide range of diseases. Research continues to explore and develop these strategies to improve clinical outcomes and address current therapeutic challenges. RNA studies will continue to be a dynamic and rapidly evolving field. Collaboration between scientists, clinicians and industry will be essential to translate basic discoveries into practical clinical applications, thus improving human health, for this reason it is essential to continue investing in this field to fully exploit its possibilities.
